# Case report: Antenatal diagnostic of a polymalformative syndrome due to biallelic BRCA2 mutations

**DOI:** 10.1002/ccr3.4838

**Published:** 2021-09-22

**Authors:** Aude Anquetil, Suonavy Khung Savatovsky, Laurent Gavard, Anne Bazin, Fabien Guimiot, Christele Dubourg, Laurent Mandelbrot, Olivier Picone

**Affiliations:** ^1^ Assistance Publique‐ Hôpitaux de Paris Service de Gynecologie Obstetrique Hôpital Louis Mourier Colombes France; ^2^ Université de Paris Paris France; ^3^ Assistance Publique‐ Hôpitaux de Paris Unité Fonctionnelle de Fœtopathologie Hôpital Robert‐Debré Paris France; ^4^ Service de Génétique Moléculaire et Génomique CHU Rennes France; ^5^ CNRS IGDR UMR 6290 Univ Rennes Rennes France

**Keywords:** BRCA2, Fanconi anemia, Genetics, Genetic counseling, Polymalformative symdrome, Prenatal diagnosis, ultrasound, VACTERL‐H

## Abstract

Testing the partner of a BRCA2 carrier must always be discussed. If both members of the couple are BRCA2 carriers, they should be informed about the high risks of polymalformative syndromes.

## Graphical abstract

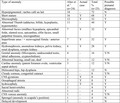

**What is already known about this topic**

Individuals heterozygous for *BRCA2* pathogenic mutations have an increased risk of inherited breast and ovarian cancer. *BRCA2* gene mutations also are the genetic basis of disease in a small proportion of children with Fanconi anemia.

**What does this study add**

Our case illustrates that testing the partner of a BRCA2 carrier must always be discussed. This must be undertaken by experienced geneticists as there is potential to generate harm and uncertainty. If both members of the couple are BRCA2 carriers, they should be informed about the high risks of polymalformative syndromes.

## 1 CASE REPORT

Individuals heterozygous for *BRCA2* pathogenic mutations have an increased risk of inherited breast and ovarian cancer. *BRCA2* gene mutations also are the genetic basis of disease in a small proportion of children with Fanconi anemia.

We present a very rare case of antenatal diagnosis of a polymalformative syndrome (VACTERL‐H) due to compound heterozygous mutations in the *BRCA2* gene. Prenatal diagnosis of those mutations has strong implications including appropriate care and genetic counseling in the family.

Individuals heterozygous for *BRCA2* pathogenic mutations have an increased risk of inherited breast and ovarian cancer.^1^, ^2^

A 39‐year‐old gravida 2 para 1 woman (she delivered two years before by caesarian section of a 2700 g healthy baby), was referred to our prenatal diagnosis center at 13 weeks of gestation (WG) for a suspected anomaly detected at the first‐trimester ultrasound examination at 11 WG +3 days. The father was a known carrier of a *BRCA2* gene mutation, which was tested following a diagnosis in his mother for ovarian cancer. The couple was not consanguineous.

We confirmed a normal 1.3‐mm nuchal translucency and a 2 × 8 mm intra‐abdominal anechogenic mass. First‐trimester serum markers screening for trisomy 21 was not performed, but a noninvasive prenatal test (NIPT) performed at her request at 17 WG was negative for trisomies 13, 18, and 21.

**FIGURE 1:**
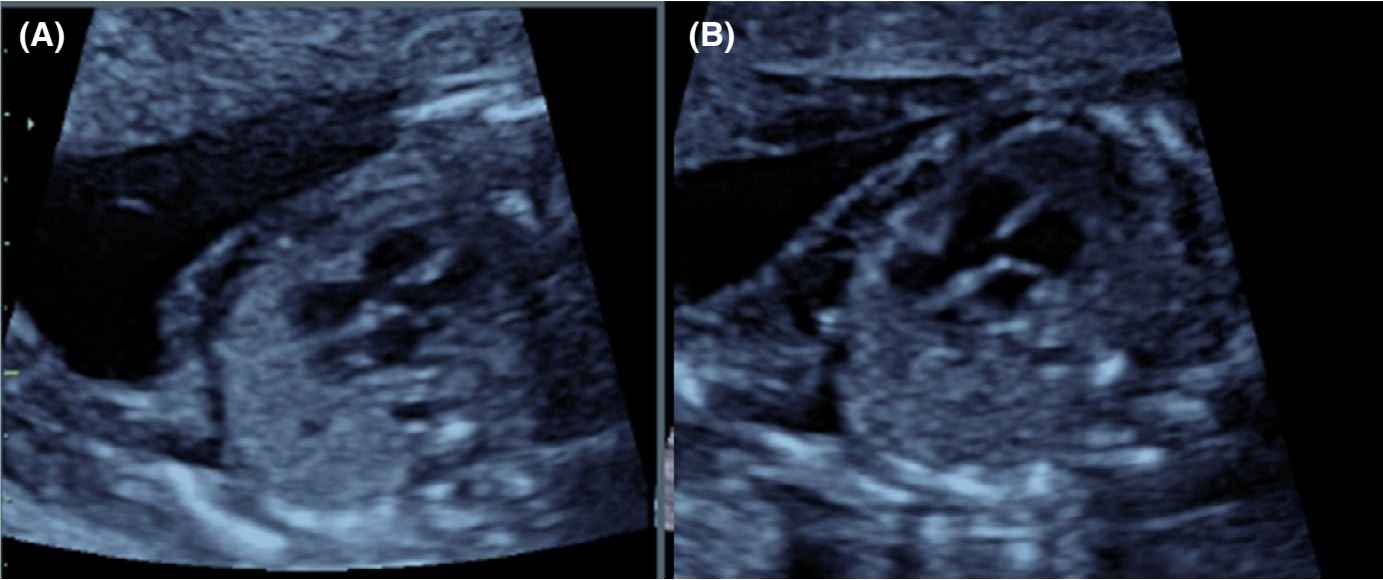
Ultrasound examination at 18 WG. (A) Suspected interventricular communication. (B) Common arterial trunk

At 18+2 WG ultrasound examination showed severe intrauterine growth restriction, cerebral ventriculomegaly, absence of septum pellucidum, cerebellar hypoplasia, and a common arterial trunk (CAT; Figure 1).

An amniocentesis was performed. The fetal CGH array was found normal (arr [X,Y] × 1,(1–22) × 2. The poor fetal prognosis was discussed, and the patient was requested for the termination of pregnancy (TOP), which was accepted according to French law, which was performed at 24 WG +1 day.

**FIGURE 2:**
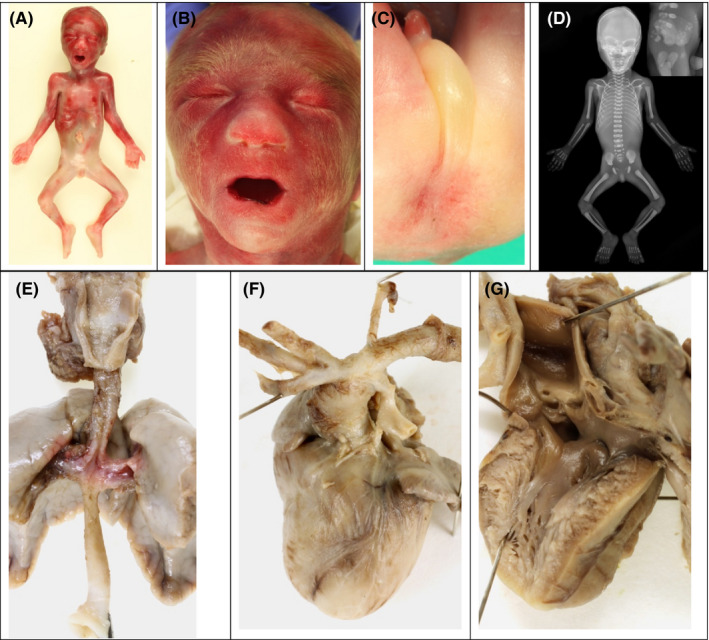
Postmortem external anomalies and X‐Ray. (A) Whole body photograph: thin fetus with normal limbs. (B) Fetal dysmophy related to the brain malformation. (C) Imperforate anus and normal male genitalia. (D) X‐rays showing growth retardation, fused vertebrae, and pelvic calcifications. The limbs are normal. (E) Esophageal atresia with distal fistula to the carina. (F) Pulmonary atresia mimicking a common arterial trunk. (G) Outlet of the left heart ventricle: ventricular septal defect

**FIGURE 3:**
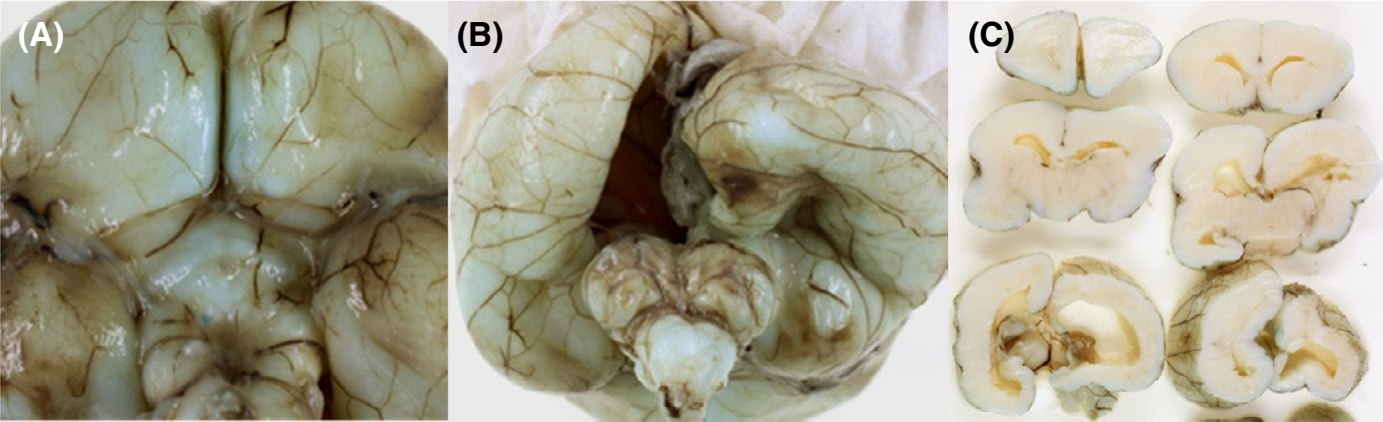
Postmortem examination of the brain. (A) Agenesis of the olfactory bulbs. (B) Cerebellar hypoplasia. (C) Coronal slices of the brain showing moderate ventriculomegaly and delayed Sylvian fissure operculization. The anterior portion of the corpus callosum is present

The fetal postmortem examination revealed multiple malformations (Figures 2 and 3).

The fetus had dysmorphic features, with enophthalmos short palpebral fissures, a wide nose with small nostrils, and a narrow philtrum. The male external genitalia was normal but the anus was imperforate. Upper and lower limbs were normal. X‐ray examination showed slight midfacial hypoplasia, normal semicircular canals, fusions of the fourth and fifth vertebral bodies, and pelvic calcifications corresponding to the sigmoid colon. Dissection of the internal organs found dilated sigmoid colon and rectal atresia. No fistula between the urinary and digestive tracts was found. The kidneys were small with a normal architecture but large ureters. The tail of the pancreas was missing. There was esophageal atresia with distal fistula to the trachea.

The heart showed pulmonary atresia with ventricular septal defect and a right aortic arch.

Brain anomalies were: micrencephaly, hypoplastic cerebellum, delayed cortical brain folding, arhinencephaly, hypoplastic pituitary, and hypoplastic optic chiasm. Coronal slices of the brain showed moderate cerebral ventricular dilatation, an enlarged inter‐thalamic adhesion, and a narrow cerebral aqueduct. The corpus callosum showed an incomplete splenium as histology demonstrated posterior Probst bundles. The hydrocephalus was related to a mild form of holoprosencephaly with signs of hypopituitarism (small testes and hypoplastic adrenal glands).

Considering these findings, the diagnosis of VACTERL‐H phenotype was proposed.

Trio exome sequencing was performed using the Agilent SureSelect Exome V7 preparation kit and an Illumina NextSeq sequencer. It revealed two compound heterozygous variants in the BRCA2 gene, one frameshift (NM_000059.3:c.5213_5216del, p. [Thr1738Ilefs*2]) inherited from the mother and a splice one (NM_000059.3:c.7007G>C, p.?) inherited from the father. These two variants are classified as pathogenic in the ClinVar database and are responsible for the polymalformative syndrome in the fetus.^2^, ^3^, ^4^ No other relevant variant was identified in the fetus.

## 2 DISCUSSION

The association of malformations in this fetus led to the diagnosis of a VACTERL‐H syndrome.^5^ 10% of patients with FA have at least three of the component parts of the VACTERL‐H association.^6^ FA is a recessive autosomal condition involving bone marrow failure, congenital malformations (skeletal defects, especially radial ray aplasia, small stature, renal and genital malformations, microcephaly with microstomia, and microphthalmia), and a predisposition to malignancy.

The distribution of the genotypes of the patients with FA VATER differs from those in the general FA population phenotype with more extensive congenital anomalies and a high incidence of pigmentary anomalies.^7^ FA with biallelic mutations *FANCD1*/*BRCA2* is associated with poorer outcomes^5^ and the frequency of VATER in such cases is estimated at 19%.^1^ Therefore, testing for *BRCA2* pathogenic mutations should be performed for every Fanconi syndrome.

A single case reported in the literature^1^ had a prenatal diagnosis of a polymalformative syndrome with hydrocephalus, fused kidneys, and growth restriction. At birth, intrauterine growth restriction was confirmed, and corneal opacities, an anteriorly placed anus, small kidneys, and long thumbs with increased laxity were observed. Table 1 reports anomalies reported antenatally and postnatally by Alter et al and in our case. The chromosome breakage test for Fanconi anemia was not performed until the age of 20 months. Direct DNA sequencing showed biallelic mutations in BRCA2, thereby identifying it as belonging to group FA‐D1.^1^ In the case of biallelic mutations in *BRCA2* affected individuals are at extremely high risk for hematologic malignancy at an early age (cumulative incidence of 41% at 5 years vs. 1% at 5 years in non‐BRCA2 FA patients)^7^ and at additional risk of solid organ tumors.^8^ Spontaneous chromosome breakage is also frequently observed.^9^, ^10^

**TABLE 1:** Types of anomalies reported prenatally and postnatally in case of biallelic mutations of BRCA2

Type of anomaly	Alter et al.^2^ (*n* = 24)	Actual case	Total % (*n* = 25)	Possible prenatal diagnosis
Hyperpigmented; taches café au lait	18	–	72	N
Short stature	16	Y	68	Y
Microcephaly	12	Y	52	Y
Abnormal Thumb (adducts, bifid, hypoplastic, hypermobile)	11	–	44	Y
Abnormal facies (midface hypoplasia, epicanthal folds, slanted eyes, epicanthus, elfin facies, small palpebral fissures, micrognathia)	7	Y	32	Y
Imperforate anus/+ rectovaginal fistula/anterior anus	7	Y	32	Y
Hydronephrosis, anomalous kidneys, pelvic kidney, renal dysplasia, and ectopic kidney	6	Y	28	Y
Genital anomaly (micropenis, undescended testis, labial adhesions, cryptorchidism)	4	–	16	Y/N
Abnormal hearing, small ear, deaf	3	–	12	Y/N
Cardiac anomaly (patent foramen ovale, ventricular septal defect)	3	–	12	Y/N
Dislocated hips, hip dysplasia	3	–	12	N
Cloudy corneas, congenital cataract	2	–	8	Y/N
CNS gyrations	1	Y	8	Y
esophageal atresia	1	Y	8	Y
hydrocephaly	1	Y	8	Y
Sacral hemivertebra	1	–	4	Y
Abnormal radii	1	–	4	Y
CNS venous anomaly	1	–	4	Y/N
Sprengel (anomaly in scapula's position)	1	–	4	N
Delayed development	1	–	4	N

The rarity of FA due to biallelic *BRCA2* pathogenic mutations supports a fundamental role of BRCA2 for prevention of malignant transformation during development. For some populations, the number of FA patients with biallelic *BRCA2* disruption is smaller than that expected from the carrier frequency; this implies that some pregnancies with biallelic *BRCA2* pathogenic mutations do not go to term. It might be pertinent to explore the *BRCA2* mutation carrier status in the couple with recurrent miscarriages who are from populations with high *BRCA2* pathogenic mutation carrier frequencies.^11^

Our case illustrates that testing the partner of a BRCA2 carrier must always be discussed. This must be undertaken by experienced geneticists as there is potential to generate harm and uncertainty.^11^ If both members of the couple are BRCA2 carriers, they should be informed about the high risks of polymalformative syndromes.

Preimplantation genetic diagnosis (PGD) must also be discussed.

In the absence of PGD, prenatal diagnosis should be offered, preferably with chorion villus sampling.

Recently, advances have been made in the field of noninvasive prenatal diagnosis where rare fetal extravillous trophoblasts circulating in maternal blood can be used for prenatal diagnosis. As described for *CFTR* variants, a similar approach could be considered for *BRCA2* variants and proposed to this family.^8^, ^12^

Genetic counseling should allow the couple to decide whether to be informed of the result in case of a single BRCA2 muted allele in the fetus, since this would not result in any malformation, but would lead to a risk of cancer later in life. If only one parent is known to be a carrier of BRCA2 pathogenic mutation, diagnostic ultrasound should be offered. In case of prenatal diagnosis of a VATER syndrome in association with intrauterine growth restriction, Fanconi anemia should be considered and a search for BRCA mutations should be carried out because that can greatly modify the future management of pregnancy and the parents' own risk of cancer.

In summary, we present a case of polymalformative syndrome due to compound heterozygous pathogenic mutations in the BRCA2 gene. Prenatal diagnosis of these mutations in such genes has strong medical implications including appropriate care and genetic counseling in the family.

## CONFLICT OF INTEREST

None declared.

## AUTHOR CONTRIBUTIONS

AA wrote the manuscript. SKS and FG performed the foetopathologic examination. LG performed ultrasound scan. AB was the genetic counseling of the couple. CD performed genetic analysis. LM was the head of the department and reviewed the manuscript. OP managed the patient and wrote the manuscript. All authors approved the final version of the manuscript.

## Data Availability

The data that support the findings of this article are available from the corresponding author upon reasonable request.
